# Bioprospecting Indigenous Marine Microalgae for Polyunsaturated Fatty Acids Under Different Media Conditions

**DOI:** 10.3389/fbioe.2022.842797

**Published:** 2022-03-17

**Authors:** Priyanshu Jain, Amritpreet Kaur Minhas, Sadhana Shukla, Munish Puri, Colin J. Barrow, Shovon Mandal

**Affiliations:** ^1^ TERI Deakin Nanobiotechnology Centre, Sustainable Agriculture Division, The Energy and Resources Institute, New Delhi, India; ^2^ School of Life and Environmental Sciences, Deakin University, Geelong, VIC, Australia; ^3^ Medical Biotechnology, College of Medicine and Public Health, Flinders University, Adelaide, SA, Australia

**Keywords:** α-linolenic acid, indigenous microalgae, media stress, polyunsaturated fatty acids, growth kinetic

## Abstract

Marine microalgae produce a number of valuable compounds that have significant roles in the pharmaceutical, biomedical, nutraceutical, and food industries. Although there are numerous microalgal germplasms available in the marine ecosystem, only a small number of strains have been recognized for their commercial potential. In this study, several indigenous microalgal strains were isolated from the coast of the Arabian Sea for exploring the presence and production of high-value compounds such as polyunsaturated fatty acids (PUFAs). PUFAs are essential fatty acids with multiple health benefits. Based on their high PUFA content, two isolated strains were identified by ITS sequencing and selected for further studies to enhance PUFAs. From molecular analysis, it was found both the strains were green microalgae: one of them was a *Chlorella* sp., while the other was a *Planophila* sp. The two isolated strains, together with a control strain known for yielding high levels of PUFAs, *Nannochloropsis oculata*, were grown in three different nutrient media for PUFA augmentation. The relative content of α-linolenic acid (ALA) as a percentage of total fatty acids reached a maximum of 50, 36, and 50%, respectively, in *Chlorella* sp., *Planophila* sp., and *N. oculata*. To the best of our knowledge, this is the first study in exploring fatty acids in *Planophila* sp. The obtained results showed a higher PUFA content, particularly α-linolenic acid at low nutrients in media.

## Graphical Abstract

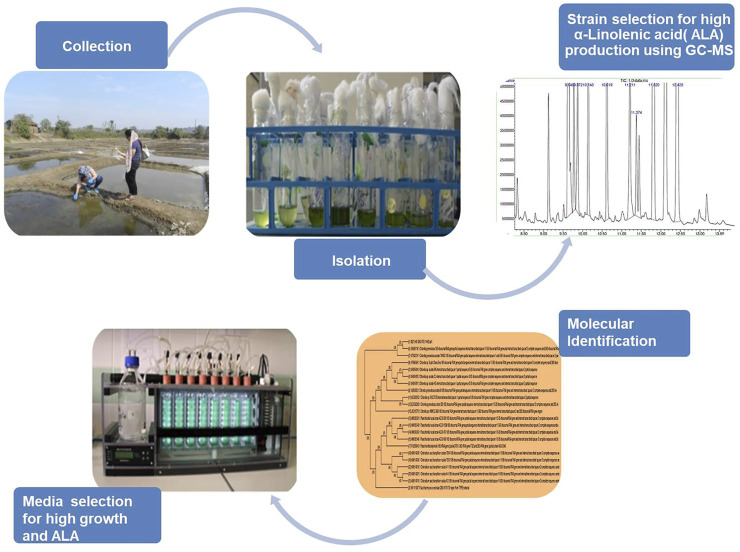

## Highlights

➢ Microalgae feedstock is the key to eco-friendly and sustainable PUFA production.
➢ Indigenous microalgal strains rich in ALA were isolated and identified from the Arabian Sea coast.
➢ Media with a low level of nutrients and salinity favors ALA enrichment.

## Introduction

Poly unsaturated fatty acids (PUFAs) and monounsaturated fatty acids (MUFAs) are essential bioactive compounds with multiple health benefits (Rincón-Cervera et al., 2022). PUFAs, particularly eicosapentaenoic acid (EPA) and docosahexaenoic acid (DHA), have tremendous applications in a variety of inflammatory conditions, such as arthritis, Alzheimer’s disease, and lupus (Yates et al., 2014). The common sources of these FAs are nuts, fishes, seeds (Ferreira-Dias et al., 2022), and organisms from the deep-sea ecosystem (Svetashev, 2021). Traditionally, marine fish are the most conventional source of PUFAs (Bagul and Annapure, 2021). However, due to declining fish stocks and the presence of contamination such as methyl mercury, dioxins, and polychlorinated biphenols (PCBs), alternative sources are required (Ruiz-Rodriguez et al., 2010). In addition, vegetarian consumers prefer algal oil to fish oil. Microalgal oils often exhibit simpler fatty acid profiles and possess a varying ratio of PUFAs with inherent antioxidant properties to protect the oils against oxidation. Marine organisms such as *Schizochytrium*, *Ulkenia*, and *Crypthecodinium* are grown heterotrophically for commercial production of DHA, particularly for uses such as infant formula where low levels of EPA are desired (Barclay et al., 1994; Ren et al., 2010; Klok et al., 2014). On the other hand, common EPA-producing algae are *Nannochloropsis*, *Nitzchia*, and *Phaeodactylum tricornutum* (Spolaore et al., 2006).

Algae are of vital importance in the primary establishment and maintenance of aquatic and marine ecosystems (Beetul et al., 2016; Barkia et al., 2019). The marine environment comprises diversity of organisms which are potential sources of bioactive, secondary metabolites, with application in pharmaceuticals, nutraceuticals, and functional foods (Barkia et al., 2019). Indigenous microalgal isolates collected from water bodies at diverse geographical locations are potential contenders for high-value compounds, such as PUFAs, and as biofuel feedstock (Maneechote et al., 2021). Moreover, accumulation of high-value compounds can be enhanced using different growth conditions, such as under stress, providing efficient cost-effective production of some metabolites (Chua et al., 2020). Enhancement of lipid and pigment productivity from the same biomass under numerous rate limiting conditions is commonly practiced (Minhas et al., 2016a). For instance, factors such as light intensity, altered photoperiod, and concentration of nutrients highly affect the microalgal growth (Parmar et al., 2011; Minhas et al., 2020). Significant diversity of microalgal isolates occurs at different geographical locations because of different nutrient variability and diverse climatic conditions (Bernal et al., 2008). Depending on the habitat and climatic conditions, microalgal isolates are known to be rich in different types of lipids, hydrocarbons, proteins, and other components (Chisti, 2007).

Microalgae are rich reservoirs of PUFAs, proteins, lipids, polyphenols, minerals, vitamins, etc. (Brown et al., 2014). Fatty acids, protein, and pigments are the most commonly available products from microalgae in market (Nagi et al., 2021). Fatty acids obtained from microalgae are applied as sustainable synthetic dietary alternatives to fish oil and possess potential in the treatment, prevention, and management of some physiological anomalies (Beetul et al., 2016). Advantages of fatty acids from microalgae over those from fish oil are primarily related to renewable and economical production (Ray et al., 2022), but they can also have tailored levels of different PUFAs that show benefit against inflammation and cardiac-related diseases such as hypertension and thrombosis (Nauroth et al., 2010; Adarme-Vega et al., 2014).

In this study, a bioprospecting pipeline, targeting microalgae along the west coast of India, was developed, and their potential for the production of PUFAs was investigated. The coast was targeted due to suitable climatic and environmental conditions, ideal for spawning and nurturing the marine life (Kamat et al., 2020). The coast has, thus, been explored extensively by numerous researchers for studying microorganisms, especially microalgae, for a wide range of potential uses (Damare et al., 2021). The objective of this work was to isolate and identify indigenous microalgal strains with high PUFA contents and to characterize the isolates by ITS sequencing. The primary selection criterion was an ability to produce high amounts of ALA. Based on high ALA levels, isolates of interest were cultivated in three different media for the assessment of PUFA productivity and fatty acid profiles. ALA productivity was optimized for the media that produced the highest ALA levels, and so this study provides useful strain, media, and growth condition information useful for enhancing PUFA levels in these microalgae.

## Material and Methods

### Collection and Isolation of Microalgae

Water samples were collected from diverse habitats ranging from the marine, backwater, and salt pans of western India, Goa (15° 32′ 0.2904″ N 73° 45′ 53.8344″ E) which possesses a coastline of 101 km (Supplementary Figure S1). The samples were collected in April 2018 from various sites during the daytime, when the Sun was overhead; the samples were put in a plastic container marked with their collection site names. Next day, samples were brought to the laboratory, centrifuged, and immediately transferred to artificial seawater media (ASW) (Andersen, 2005) at 25°C with 150 rpm orbital shaking with a photoperiod of 16 h of light (100 μmol/m^2^/s) alternating with 8 h of darkness, up to the late exponential phase (Minhas et al., 2016b).

To segregate large population and to obtain maximum isolates from the collected water samples, the standard dilution plating method was followed. After 2 weeks, serial dilution up to 10^5^ and 10^6^ was performed on sterile ASW agar plates (1.6% w/v), and samples were incubated until colonies appeared. Individual colonies were transferred axenically in liquid ASW medium and were observed under the microscope (Carl Zeiss, Germany). Morphologically non-identical strains were selected for further study.

### Microalgal Strains

Isolated strains were grown in a media broth in 100-ml Erlenmeyer flask containing 50 ml of ASW medium (Lee et al., 2014) at pH 8. The experiments were conducted in a temperature-controlled growth chamber at 25°C under a photoperiod of 16:8 (light:dark) at a light intensity of 120 μmol/m^2^/sec. In order to attain high PUFA-containing isolates, the freeze dried biomass from the stationary phase cultures was processed through lipid extraction and fatty acid analysis, as described in the following analytical methods. All the salts and chemicals used in this study were of analytical grade and procured from Sigma-Aldrich and Merck Chemicals.

### Identification of Microalgal Strains

The potential candidate displaying maximum levels of PUFAs was selected further for yield enhancement studies and was subjected to ITS sequencing by implying ITS1 and ITS4 primer sets. ITS sequencing was performed at Eurofins, Bangalore, India, for species identification.

#### DNA Extraction

The total genomic DNA of the algal isolate was isolated and purified using the DNA extraction kit (NucleoSpin^®^). About 1.5 ml of the exponentially grown algal culture was centrifuged at 7,500 g for 8 min at 4°C. The resulting cell pellet was lysed using liquid nitrogen and was further dissolved and mixed in 140 μL buffer T1 and 8 μL proteinase K solution. The mixture was left at 56°C for 1 h incubation in a thermomixer (Thermomixer comfort, Eppendorf, New Delhi, India) for complete cell lysis. Thereafter, B3 buffer amounting to 140 μL was added to the same vial and left at 70°C for 5 min incubation in a thermomixer. Samples after attaining room temperature were centrifuged for 5 min at 9,000 × g, and the obtained supernatant was transferred to a new microcentrifuge tube. Absolute ethanol of 140 µL was added to the samples; immediately after the addition of ethanol, a thread-like precipitate appeared. The vials were then left at −20°C for 15 min for complete precipitation. The obtained precipitate was transferred to a NucleoSpin^®^ tissue column, and a collection tube was placed below it. Centrifugation was performed for all samples for 2 min at 10,000 × g; all algal samples were eluted separately. The column was placed again in the same collection tube, and 100 μL of washing buffer W1 was added to the same vial; samples were centrifuged for 1 min at 10,000 × g, and the process was repeated again for proper washing. Finally, a NucleoSpin^®^ tissue column was placed in a new 1.5 ml microcentrifuge vial, and 30 µL elution buffer (BE) was added directly onto the center of the column for eluting DNA, which was centrifuged for 2 min. Extracted DNA was kept at −20°C for further analysis.

#### PCR Amplification

DNA fragments of selected strains were observed on the gel *via* gel electrophoresis with respect to the 1 kb DNA ladder (GeneDireX, Taiwan, China). The DNA concentration obtained from microalgae was between 70 and 92 ng/μL. The obtained DNA was subjected to PCR amplification; PCR was carried out using 0.1 mM dNTPs, 10 pmol of each primer, 1 U of Taq DNA polymerase, and the supplied reaction buffer (Biotools, Madrid, Spain) in the total volume of 25 μL. Each reaction was performed in duplicates in a T-100 thermal cycler (Bio Rad Laboratories Inc., California, United States) under the following conditions: initial denaturation at 95°C for 5 min, followed by 35 cycles at 94°C for 35 s, 60°C for 1 min, 72°C for 1 min, followed by a final extension period at 72°C for 10 min, and rest at 4°C. Agarose gel electrophoresis was performed which depicted sharp bands of algal PCR products at 600 bp. Sanger sequencing was performed for both the samples at the commercial facility service of Eurofins Private Limited (Bangalore, India). The sequences were subjected to BLAST (BLASTN, NCBI) analysis, and their homology was established.

### Study on the Effect of Different Media on Growth and Fatty Acid Profiling

Isolates from different sites were selected (one isolate per site) based on the highest PUFA content. The selected isolates were then grown in three different media (Andersen, 2005), viz., modified F/2 (Guillard et al., 1975), MASM (https://www.ccap.ac.uk/wp-content/uploads/MR_MASM.pdf), and MKM Watanabe, A. (1960) in a multicultivator (MC 1000-OD, Photon Instrument Systems, Drasov, Czech Republic). The isolates with an initial cell count of 3 × 10^6^ cells/mL calculated using a Neubauer hemocytometer (Rohem Instruments, Nashik, Maharashtra, India) were inoculated in the 70 ml of media in multicultivator tubes having a volume of 120 ml under photoautotrophic conditions with a light intensity of 120 μmol/m^2^/sec and a temperature of 25 ± 1°C, with a 16:8 h (L:D) photoperiod (Minhas et al., 2020). The aeration rate was maintained at 0.12 ml/min. Growth of microalgae was examined by measuring the daily changes in the optical density (OD) at 680 nm by OD viewer software attached to the cultivator. The microalgal growth rate of isolates was determined by fitting the OD at the exponential phase of each isolates to the late stage exponential growth phase (Wang et al., 2010). Once the isolates achieved their optimum growth, at the late stationary phase on an average, they were harvested by centrifuging them at 7,000 rpm for 10 min at 4°C followed by lyophilization. The samples were further subjected to GC-MS for fatty acid profiling. Studies were performed in sets of triplicates.

### Analytical Methods

#### Lipid Extraction and Fatty Acid Analysis

Total lipids were extracted from the freeze dried biomass by adopting the method developed by Lewis et al. (2000) with some modifications. In brief, 3 ml solution of chloroform:methanol (2:1, v/v) was added to 10 mg of the dried algal biomass and was homogenized by using a vortex shaker (Spinix, Maharashtra, India) for 2 min followed by centrifuging for 15 min at 10,000 × g. The process is repeated three times for complete extraction until a colorless biomass is achieved. The obtained fractions were pooled, and water was added; the upper layer containing methanol and water was discarded. The chloroform fraction was passed through syringe filters and was transferred in a pre-weighed glass vial. The vials containing lipids were incubated in a hot air oven for 6–7 h at 50°C. Finally, the total lipids were measured gravimetrically.

#### Preparation of Fatty Acid Methyl Esters

FAME profiles were determined using the method described by Christie (1987) (Christie, 1982). For preparation of FAMEs, dried lipid samples were obtained after incubation and oven drying; 500 μL of toluene was added to the sample, followed by the addition of 10 μL of the internal standard (50 mg of C19:0- methyl non-adecanoate), acetyl chloride 400 μL (prepared by adding 1 ml acetyl chloride dropwise to 10 ml of methanol on ice), and butylated hydroxytoluene 200 μL, and the samples were incubated at 50°C overnight. The following day, 1 ml of 5% NaCl and 1 ml of hexane were added to the dried samples. Finally, the solvent layer containing hexane was analyzed by GC (Agilent 122–2,332 column, Santa Clara, California, United States) equipped with mass spectrometry (MS) with capillary columns (DB-23; 30 × 0.25 mm; film thickness, 0.25 μm). Retention time of the known fatty acid standard mix (37 FAME mix, Supelco, Sigma-Aldrich) was identified, and the peaks of fatty acid chains were analyzed and quantified. ChemStation chromatography software (Agilent Technologies, Santa Clara, California, United States) was utilized further for integrating the peaks of targeted fatty acids. 1 μL volume of the sample was injected in the instrument maintained at 250°C with helium as a carrier gas. The chemicals and standard for FAMEs (C19:0) used in this study were of analytical grade and were procured from Sigma-Aldrich (St. Louis, United States).

### Statistical Analysis

All statistical analyses and graphics were performed using R (version 4.1.2). To measure the growth rate, optical density was fitted against four different growth models (Richard, logistic, Gompertz, and modified Gompertz) using the gcFitModel function of the “grofit” package. The best fit model was selected based on Akaike information criterion (AIC) values. Instead of using the popularly used logistic growth curve, different growth models were applied because of the variation of growth curves. For principal component analysis (PCA), the “factoextra” package was applied. The function “glht” in the package “multcomp” was used for Tukey’s *post hoc* test.

## Results and Discussions

### GC-MS Analysis of Isolated Microalgae for the Presence of Omega-3 Fatty Acid

The unialgal cultures established from the samples collected from diverse water bodies of Goa were subjected for GC-MS profiling and are tabularized in Table 1. Goa is identified as a suitable area for microalgae cultivation, as per reports of the National Renewable Energy Laboratory, United States (Milbrandt and Jarvis, 2010). Several species of zooplanktons (Sai Elangovan and Gauns, 2021) and phytoplanktons (Untawale et al., 1980; Raghukumar et al., 1991; Bhandari et al., 2012) are identified from their water bodies on a regular basis (Damare et al., 2021). Therefore, different water bodies, namely, Bagha (marine), Salim Ali (mangrove), NIO (marine), and Sirdao (brackish) were targeted for sample collection. The unialgal established cultures were screened for their FA profiling for estimating the contents of PUFAs present in the established strains. GC-MS profiling established the major fatty acids in all the strains including oleic acid (C18:1), linoleic acid (C18:2), and ALA (18:3). ALA (18:3) accumulated in the highest amounts in all the strains compared with the other two fatty acids, followed by linoleic acid (C18:2) and oleic acid (C18:1). Similar to this study, researchers isolated the four algal strains belonging to the family Chlorophyceae from the coastal zone of Goa. FA profiling revealed the presence of both saturated FAs and unsaturated FAs, including oleic, linoleic, and linolenic acids (Bhandari et al., 2012).

**TABLE 1:** The relative percentage of different fatty acid chains present in the isolated microalgal isolates.

S. No	Collection site and their habitat type	Strain code	Palmitoleic acid (C16:1)	Oleic acid (C18:1)	Transvaccenic acid (C18:1)n-7	Linoleic acid (C18:2)	α-Linolenic acid (C18:3)	Eicosatetraenoic acid (C20:4)	cis-11,14-Eicosadienoic acid (C20:6)	Eicosatrienoic acid (C20:3)	Tetracosanoic acid (C24:0)
1	Bagha (Marine)	BAG1	12.48	3.73	1.40	13.025	69.12	—	—	—	0.22
2	Bagha (Marine)	BAG2	-	12.13	-	40.21	45.14	—	—	—	2.48
3	Sirdao (Backwater)	SIR	-	12.20	10.27	26.95	50.52	—	—	—	0
4	Salim Ali (Backwater)	SA	5.16	13.06	6.25	34.41	36.76	5.65	—	4.28	1.51
5	Dona Paula (Marine)	NIO	3.35	14.50	-	36.33	45.62	—	0.49	—	0

The highest amount of ALA was accumulated in one of the species (BAG1) collected from the site Bagha at 69%, followed by 51% for SIR and ∼46% for NIO (Table 1). The second most abundant fatty acid for all three strains was linoleic acid (C18:2) with ∼40.2% for the isolate BAG2, followed by NIO, SA, and SIR (Table 1). The relative FA percentage of palmitoleic acid (C16:1) was the highest in BAG1 at ∼12%, followed by 5% in SA and 3% in NIO. In a study conducted by Nagappan et al., they identified a *Desmodesmus* sp. strain with the potential for production of biodiesel and omega-3 FAs (Nagappan and Kumar Verma, 2018). BAG1, having the highest amount of C18:3 and C16:1, may also find application in both biodiesel and nutraceutical production.

NIO depicted traces of eicosadienoic acid ∼0.49%, whereas eicosatrienoic acid and eicosatetraenoic acid were accumulated by SA at 6% and 4%, respectively. Isolates BAG1 and NIO were selected for further study due to their highest percentage of C18:3, at 70% and 45%, respectively. SIR was not selected, partly because of its slower growth rate (data not shown). The two selected isolates, namely, NIO and BAG1 were further evaluated for their efficiency in producing biomass and lipid yields. At the stationary phase, after 15 days of cultivation in ASW media, biomass of NIO and BAG1 reached up to 620.6 and 498.8 mg/L, respectively, whereas the lipid content of the NIO and BAG1 reached 14.5 and 14.9% of the dry weight of biomass, respectively. The two selected strains were subjected to molecular identification followed by media studies.

### Molecular Identification and Phylogenetic Analyses

The high molecular weight DNA of BAG1 and NIO strains was extracted by the NucleoSpin kit. ITS1 (TCC​GTA​GGT​GAA​CCT​GCG​C) and ITS4 (TCC​TCC​GCT​TAT​TGA​TAT​GC) were employed as the universal primers for the amplification of aforementioned strains. ITS sequencing was conducted for the molecular identification of two algal isolates (Eurofins, Bangalore, India). The obtained sequences of both the strains were subjected to BLAST (BLASTN, NCBI) analysis, and their homology was established. Results revealed NIO was identical to *Planophila* (MT991544.1), with 91.53% similarity, and the other strain BAG1 showed 98.81% similarity with *Chlorella* (MH045494.1) in the homology analysis.

**FIGURE 1:**
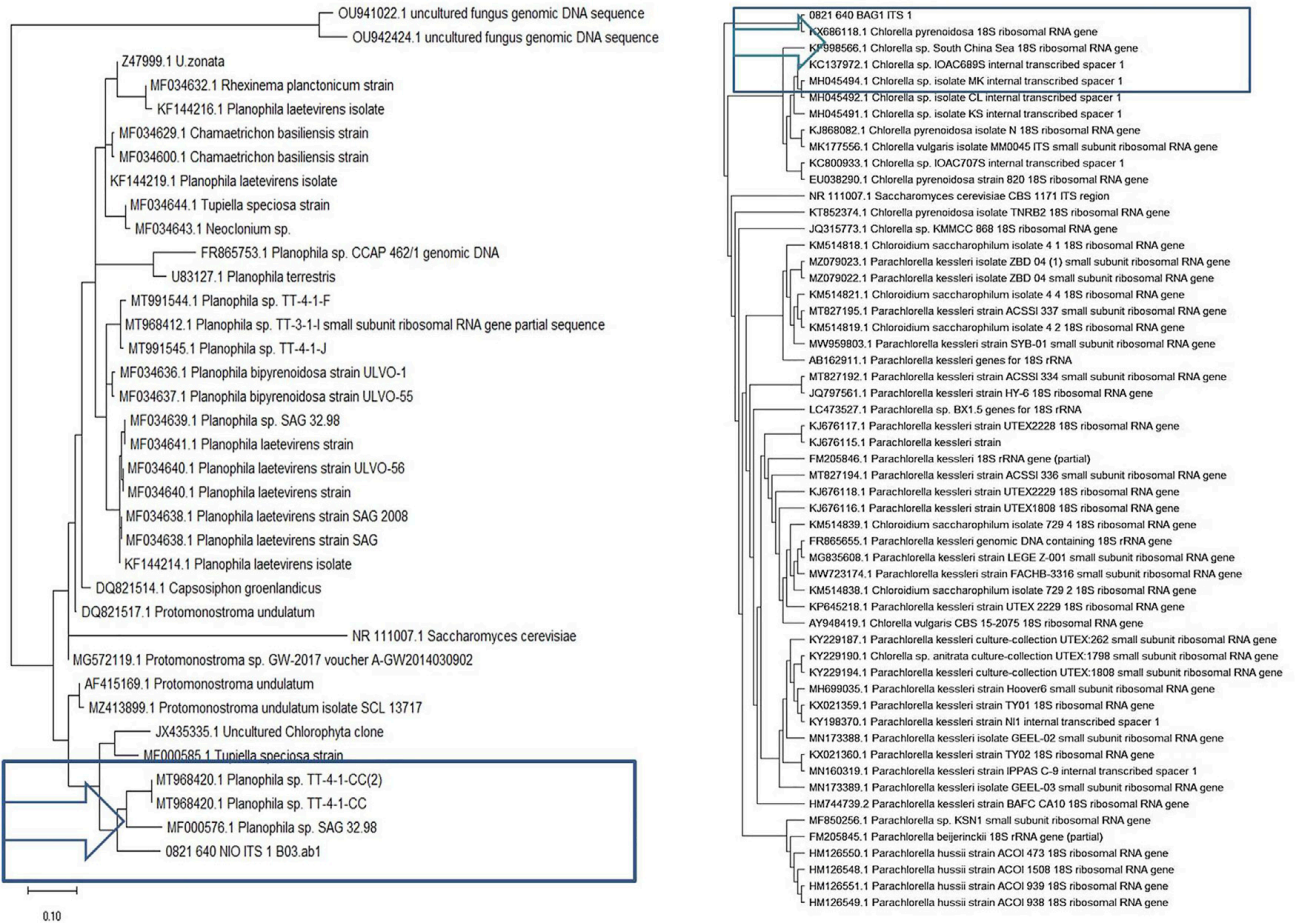
Phylogenetic tree of microalgal isolates revealing cluster grouping of **(A)** NIO ITS sequences are closely related to Planophila. **(B)** BAG1 ITS sequences are related to chlorella; Saccharomyces cerevisiae was used as out-group.

Phylogenetic analysis was conducted on the BLASTN results of both samples using maximum likelihood algorithms. Sequences were aligned with the MUSCLE (MEGAX), and a phylogenetic tree was constructed for NIO and BAG1 strains with other 50 different species of algae and with one out-group species *Saccharomyces cerevisiae*, respectively. Phylogenetic analysis showed that NIO clustered together with *Planophila* sp. (MT991544.1) Figure 1A and BAG1 with *Chlorella* (MH045494.1) Figure 1B. Thus, the results confirmed that NIO is highly identical with *Planophila* and BAG1 with *Chlorella*, respectively.

**FIGURE 2:**
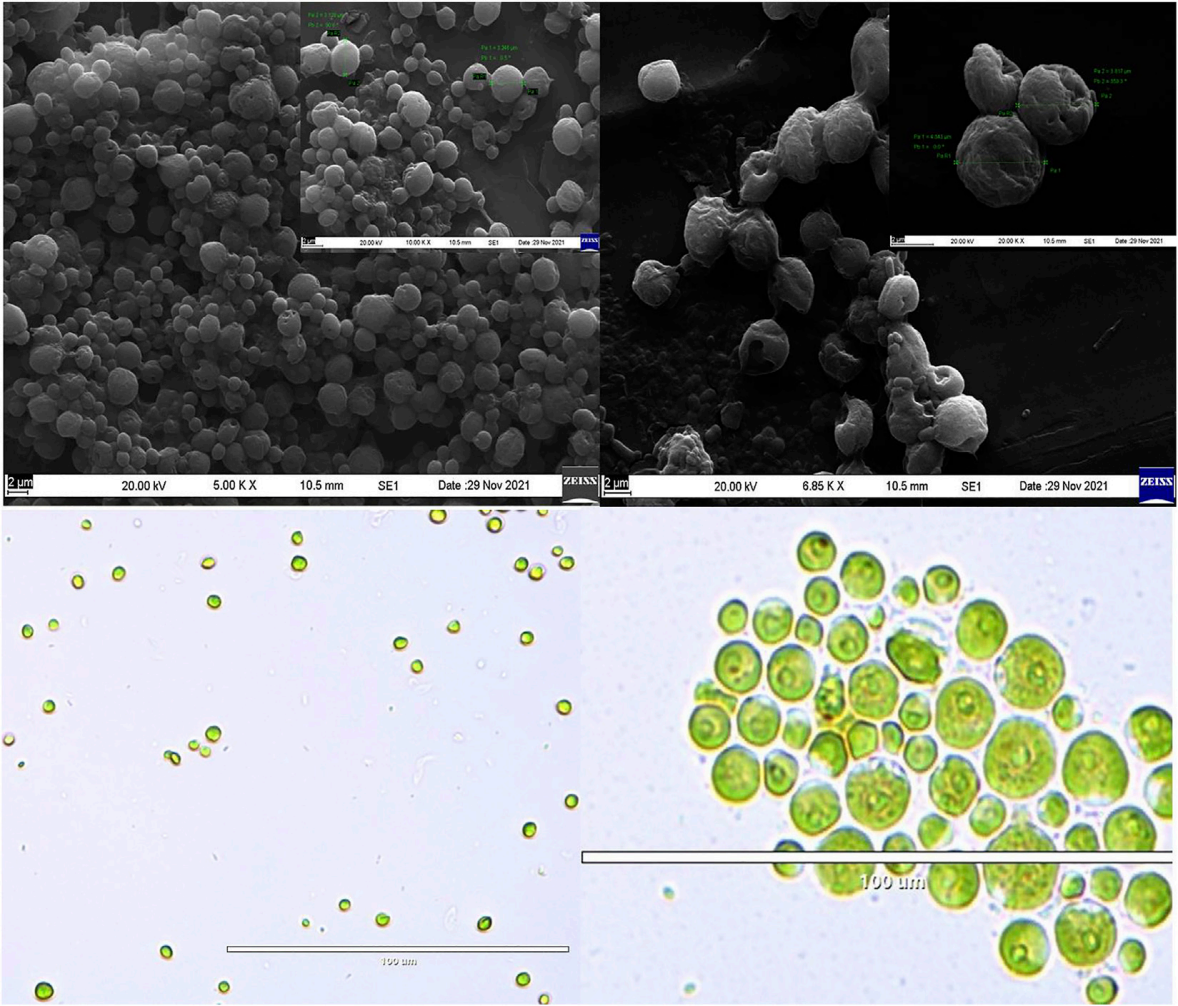
SEM images of **(A)** *Chlorella* and **(B)** *Planophila*. Light microscopic images of **(C)** Chlorella and **(D)** Planophila.

To the best of our knowledge, this is the first time the strain *Planophila* sp. was isolated from the region of Goa and is studied for fatty acid profiling in different media. However, *Planophila* sp. was previously reported to be isolated from Asian regions (Watanabe, 1983). The species were reported to be found in fresh water and soil (Szymańska and Werblan-Jakubiec, 1999; Friedl et al., 2012). The morphology of these strains is presented in Figure 2.

### Effect of Different Media on Growth and Fatty Acid Profiling

Nutrients are the key driver for microalgal growth and metabolite synthesis (Mandal and Mallick, 2009). A trade-off between growth and lipid levels has been reported in some microalgal studies (Shurin et al., 2014; Pandey et al., 2020). In this study, instead of applying the direct stress-based approach by eliminating or reducing nutrients from a particular medium, isolated *Planophila* sp. and *Chlorella* sp. were subjected to three different nutrient media, a method to elevate PUFAs. The commercial strain *N. oculata*, known for a higher PUFA content (Zanella and Vianello, 2020), was selected as a control for comparison.

**FIGURE 3:**
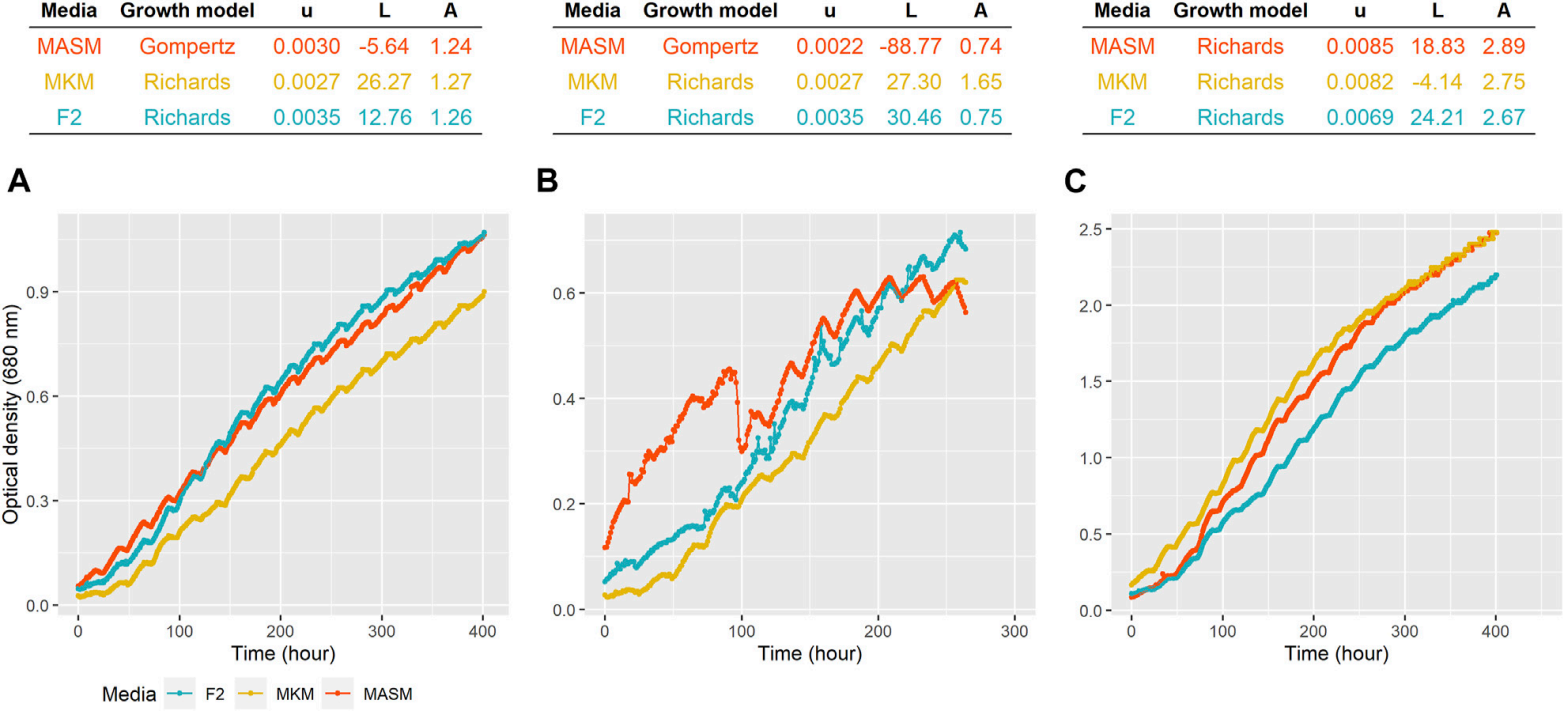
Growth of **(A)** *Planophila* sp., **(B)** *Chlorella* sp., and **(C)** *N. oculata* in different media. The top panel shows the model selected for each strain growing at different media. u = specific growth rate (hour^−1^), L = lag period (hour), and A = carrying capacity (OD at 680 nm).

**FIGURE 4:**
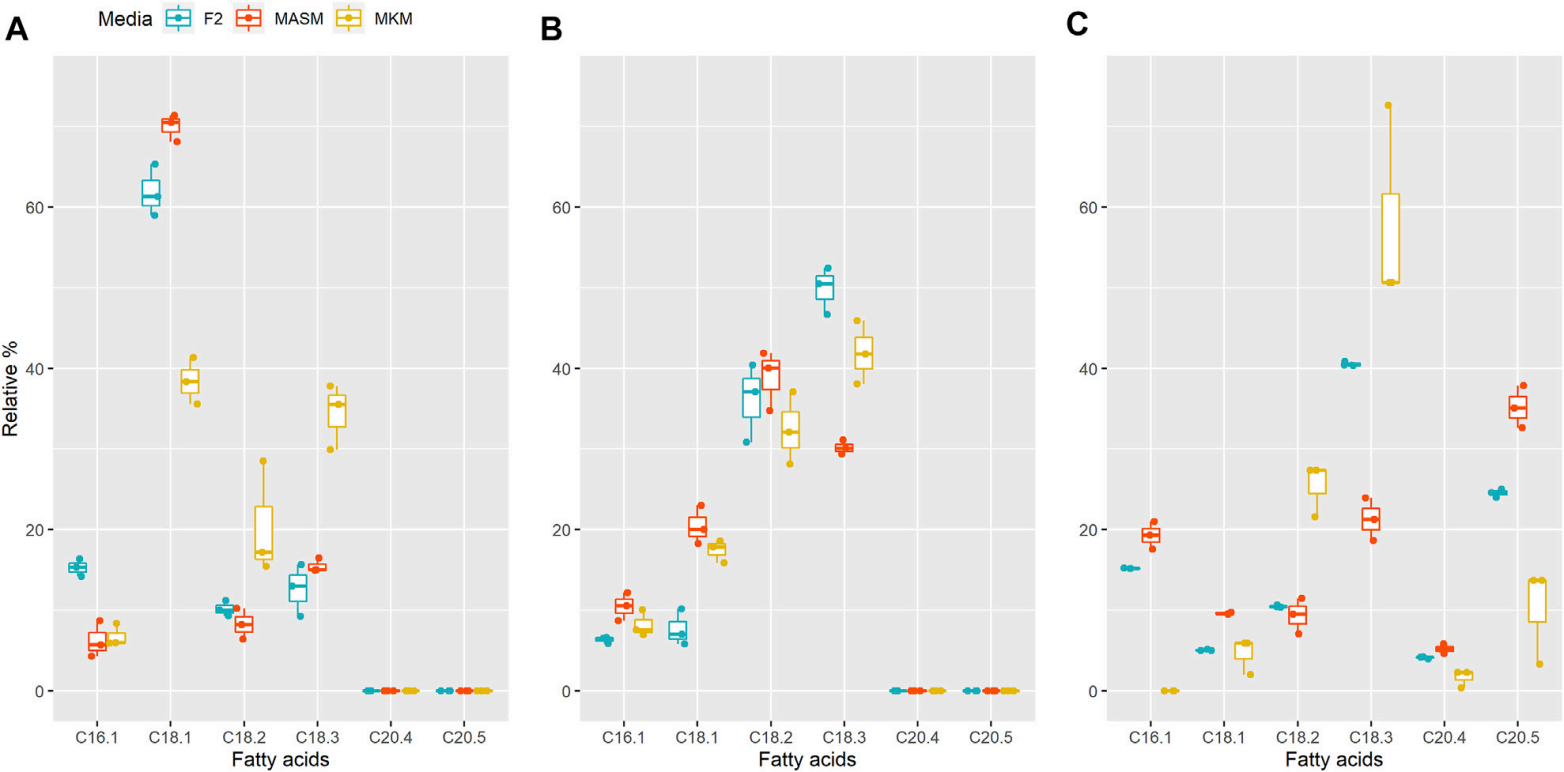
Relative FA contents in **(A)** *Planophila* sp., **(B)** *Chlorella* sp., and **(C)** *N. oculata* in different media. In each box, the middle horizontal line shows the median, the outer lines show the 25 and 75% confidence intervals, and vertical lines show 95% confidence intervals.

Four different growth kinetic models logistic, Gompertz, Richards, and modified Gompertz were applied to understand the growth dynamics attained under the different media conditions (Supplementary Figure S2). As shown in Figure 3, kinetic model Richards and Gompertz were selected as the best model based on the lowest Akaike information criterion (AIC) values. For *Planophila* sp., the highest growth is observed for F/2 and MASM media in which salinity was 30 practical salinity units (PSU), as opposed to 15 PSU in MKM. The highest growth of *Planophila* sp. in the media with higher salinity conditions indicated their selective preference towards a marine-like environment, mimicking their natural marine habitat (Pandit et al., 2017). The salinity stress affects the microalgal cells and their physiological mechanism. Parameters such as precursors, influx, and uptake of ions in and outside the cell membrane (Srivastava et al., 2014) and sodium ions and their role in photosynthesis (Salama et al., 2013) are the key factors for the changes and enhanced specific fatty acid composition (Srivastava et al., 2014). The presence of excess sodium chloride causes reactive oxygen species (ROS) formation, leading to oxidative stress and breakdown of cellular macromolecules (Chokshi et al., 2015). Another possible explanation of the lower growth rate in the MKM medium is the lower nutrient concentration which is limited in the media (Pandit et al., 2017). *Chlorella* sp. depicted highest growth in F2 media followed by MKM media. The lower growth rate in MASM in *Chlorella* sp. is due to the sudden growth depression between 80 and 90 h (Figure 4B). The control strain *N. oculata* exhibited a maximum growth in MASM media, followed by MKM and F/2. The growth of *N. oculata* is much higher and reached the carrying capacity at 2.89 (OD) which is 3–4 times than the isolated strain. These results suggest further optimization in growth parameters such as light intensity (Metsoviti et al., 2020) and temperature (Chokshi et al., 2020) may be required.

In order to combat diverse environments, specifically higher salinity conditions and low nitrogen availability, microalgae alters or enhances the PUFA production (Dhanya et al., 2020). Enhancement in the TAG content (Kan et al., 2012) and intracellular lipids in microalgae (Zhila et al., 2011) are also reported for salinity stress. For instance, Annamalai et al., reported the enhanced lipid production in one of his selected strain under lower salinity conditions (Annamalai et al., 2016). Interestingly, the results revealed that the percentage of ALA (C18:3) was the highest in media with the lowest salinity (9 g/L), amounting to 35.5% in *Planophila* sp. and 41% in *Chlorella sp.* in MKM media. A similar finding was obtained for the control strain *N. oculata* with 50% of the ALA content in the same media. Differences in the fatty acid composition among different media for a particular strain indicated that the salt type and concentrations in media impacts the fatty acid content, consistent with optimizing media, for salt can be useful for targeting levels of specific fatty acids (Minhas et al., 2016a; Haris et al., 2022).

In *Planophila* sp., the highest percentage of oleic acid at 71% occurred in MASM media, with lower levels in F/2 and MKM media, whereas linoleic (17%) and linolenic acid (35%) were maximal in MKM media (Figure 4A). Similar to *Planophila* sp., *N. oculata* showed highest linoleic acid and linolenic acid contents in MKM media (Figure 4C). However, in *Chlorella sp.* maximum accumulation of linolenic acid (50%) and linoleic acid (37%) was observed in F/2 media, followed by MKM and MASM media. The differences in types of fatty acid accumulation in different media support earlier findings in which nutrients were found to regulate FA biosynthesis (Ren et al., 2010; Dhanya et al., 2020). Thus, selection of optimum media should be carried out before attenuation of specific stress factors for enhancing targeted fatty acids. The findings of the present study in terms of highest ALA content of the two well characterized isolates are tabulated together with the other microalgae in Table 2. The ALA content found in two different strains of *Chlorella*, i.e., *Chlorella vulgaris* NIES-1269 and *Chlorella* sp. Carolina-15–2069 was 35 and 17.9%, respectively (Othman et al., 2019), whereas the same isolate in the present study was producing an ALA content about 50% in F/2 media. Therefore, explaining the variations in the ALA/PUFA productivity in similar microalgal species collected from different habitats is possibly due to their growth, media, and nutrient conditions.

**TABLE 2:** ALA content (%Total fatty acids) present in different microalgal species.

S. No	Microalgal species	ALA (% total fatty acids)	References
1	*Dunaliella primolecta*	41.1	Viso and Marty, (1993)
2	*Nannochloris sp*	28.2	Lang et al. (2011)
3	*Parietochloris incisa*	14.3	Lang et al. (2011)
4	*Nostoc commune*	38.1	Lang et al. (2011)
5	*Skeletonema costatum*	25.31	Widianingsih et al. (2013)
6	*Thalassiosira sp.*	7.93	Widianingsih et al. (2013)
7	*Isochrysis sp.*	11.57	Widianingsih et al. (2013)
8	*Acutodesmus obliquus* CN01	38	Othman et al. (2019)
9	*Chlorella vulgaris* NIES-1269	35	Othman et al. (2019)
10	*Chlorella* sp. Carolina-15–2069	17.9	Othman et al. (2019)
13	*Planophila* sp	35.5	This study
14	*Nannochloropsis oculata*	50	This study
15	*Chlorella sp.*	50	This study

**FIGURE 5:**
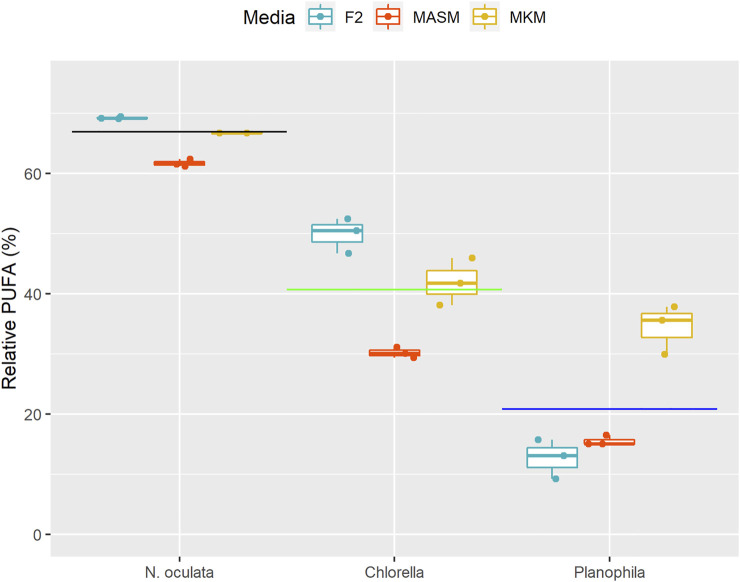
PUFA percentages of three different algal isolates grown in three diverse media. In each box, the middle horizontal line shows the median, the outer lines show the 25 and 75% confidence intervals, and vertical lines show 95% confidence intervals. The black line indicates the mean PUFA content of *N. oculata*, green for *Chlorella* sp., and blue for *Planophila* sp.

Fatty acid data was further analyzed by considering only PUFAs (Figure 5) in which only FA molecules (unsaturation >2) with a chain length of 18 or more carbon atoms were counted (Park et al., 2002). The PUFA content was significantly different in three different strains (F_2,18_ = 515.01, *p* < 0.0001, Table 3). The high PUFA content was found in *N. oculata* followed by *Chlorella* sp. and *Planophila* sp. (Tukey’s *post hoc* test, *p* < 0.05, Supplementary Table S1). Although the media have a significant effect on the PUFA content (F_2,18_ = 41.29, *p* < 0.0001, Table 3), PUFA contents were not different between MKM and F2 media (Tukey’s *post hoc* test, *p* = 0.282, Supplementary Table S2).

**TABLE 3:** ANOVA table showing the effect of strain and media on the PUFA content.

	Degree of freedom	Sum square	Mean sum square	F Value	Probability (>F)
Strain	2	9,621	4,810	515	01 < 2e-16 ***
Media	2	771	386	41.29	1.88e-07 ***
Strain: Media	4	781	195	20.91	1.44e-06 ***
Residuals	18	168	9	—	—

**FIGURE 6:**
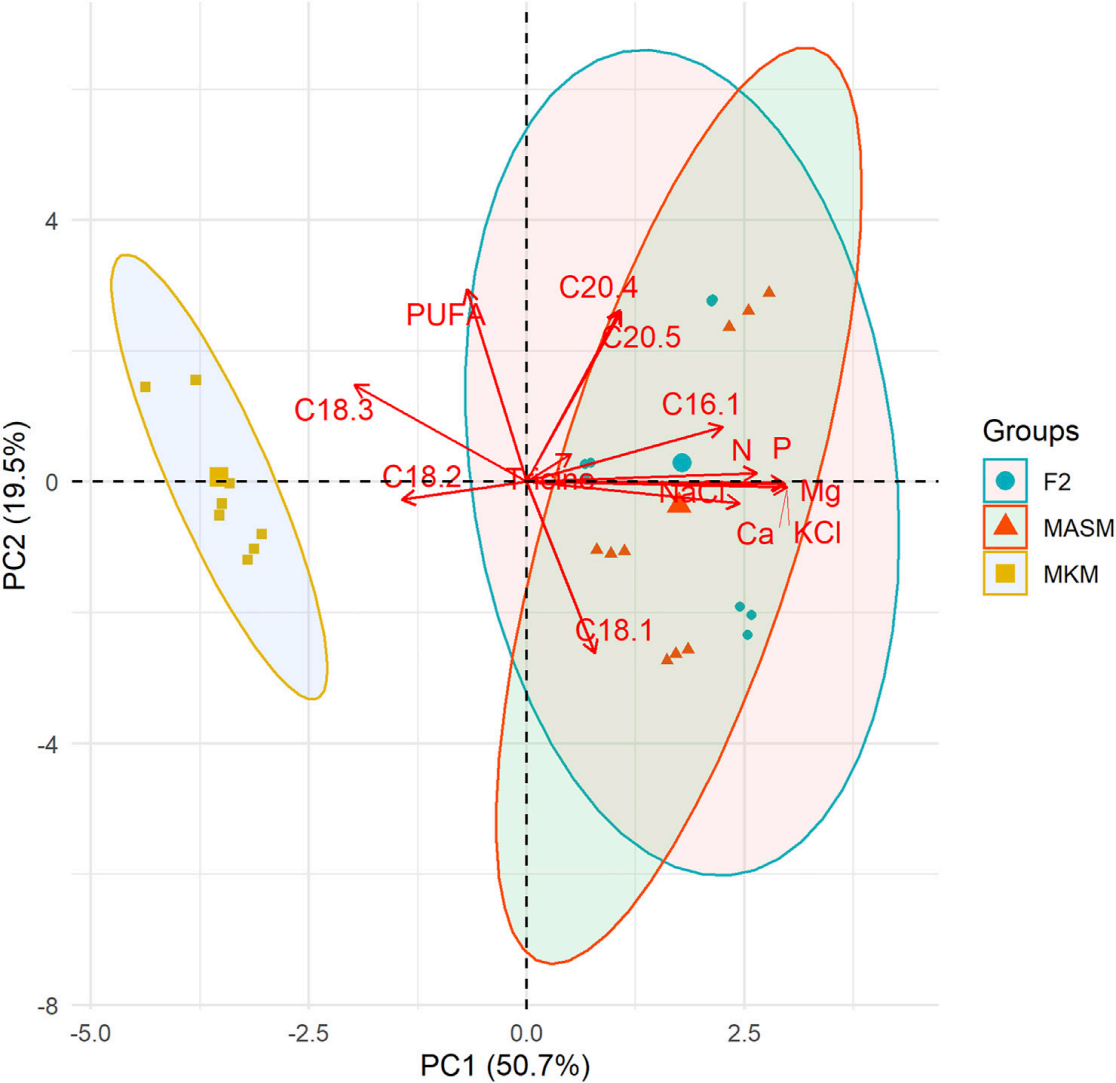
Principal component analysis (PCA) shows the correlation among nutrients contents and fatty acids of the three different strains. Each point indicates the position of the strain along PC1 and the color for media.

The principal component analysis (PCA) in Figure 6 summarizes the correlations among media components and fatty acids of the three strains. Principal component 1 (PC1) and principal component 2 (PC2) axes explained 47.4% and 24.4% of variation among strains. The overlapping between F2 and MASM indicates their similarities in media composition and FA profiling as compared to the MKM medium. The accumulation of linoleic (C18:2) and linolenic acids (C18:3) was closely associated and negatively related to the nutrient content of the media. Linolenic acid (C18:3) accumulated more in MKM media in which the nutrient concentration is comparatively low. Similar to our observation, Trommer et al. (2019) reported a decline in ALA in phytoplanktons under higher nutrient concentrations in the natural lake community. Our results agree with others that salinity had a negative correlation with ALA (Trommer et al., 2019). Overall PUFA accumulation in this study is independent of the nutrient concentration. In contrast to our study, several studies reported higher nutrient level results in increasing galactolipids which are rich in PUFAs (Guschina and Harwood, 2006; Guo et al., 2016). The close association among the nutrient components in the PCA plot is the major limitation in our study to describe the variation FA unsaturation based on each component of the media. Thus, further experiment with larger variation in nutrients of the media is recommended to establish the relation between the media component and FA unsaturation. Although, this study showed that MKM with comparatively lower nutrients could be a suitable growth media for improved FAs without compromising growth.

## Conclusion

In the present study, authors reported the isolation and identification of two microalgal isolates, *Planophila* sp. and *Chlorella* sp. collected from the west coast of India, Goa. The work demonstrated the initial screening and selection of these microalgae based on their PUFA contents, followed by their molecular characterization. Additionally, media studies employing three different media were performed to compare and analyze variations in the FA content, including levels of ALA, within the same isolates in diverse media. Furthermore, growth kinetics studies of the isolates were performed to compare the growth patterns in three different media. The results showed that media with lower nutrient levels increased the ALA content, as found in MKM media, amounting to 35.5% in *Planophila* sp. and 41% in *Chlorella* sp., respectively. The study shows that media optimization is important before attenuation of stress factors for optimizing targeted FA levels. The media studies are simple, economical, and act as a preliminary selection tool for defining the optimum physiological conditions for each indigenous algal strain. Each indigenous strain from a particular environment requires experimentation to determine optimum growth conditions since growth does not correlate directly with that of standard algal strains. Thus, targeted growth studies of indigenous strains are required to determine the potential of local organisms for the optimization of bioresource production from algae.

## Data Availability

The original contributions presented in the study are publicly available. This data can be found here: NCBI gen bank, accession numbers SUB10895695, SUB10888826.
