# Supplementary material for: Bioprospecting Indigenous Marine Microalgae for Polyunsaturated Fatty Acids Under Different Media Conditions
Source: Front Bioeng Biotechnol. 2022 Mar 17;10:842797. doi: 10.3389/fbioe.2022.842797 (PMC8971906; doi:10.3389/fbioe.2022.842797)
Supplement: Supplementary file 1 [file DataSheet1.docx]

**Supplementary Information**

Bioprospecting Indigenous Marine Microalgae for Polyunsaturated Fatty Acids Under Different Media conditions

Priyanshu Jain^1, 2^, Amritpreet Kaur Minhas^1^, Sadhana Shukla^1^, Munish Puri^3^, Colin J. Barrow^2^, Shovon Mandal^1*^

^1^ TERI Deakin Nanobiotechnology Centre, Sustainable Agriculture Division, The Energy and Resources Institute, New Delhi, India

^2^ School of Life and Environmental Sciences, Deakin University, Geelong campus at Waurn Ponds, Victoria 3217, Australia

^3^ Medical Biotechnology, College of Medicine and Public Health, Flinders University, GPO Box 2100, Adelaide 5001, Australia.

**Figure S1.** Map of sampling site on the coast of Arabian Sea. The location of sampling is shown in red colour. The locations of water sampling sites are shown with red dots.


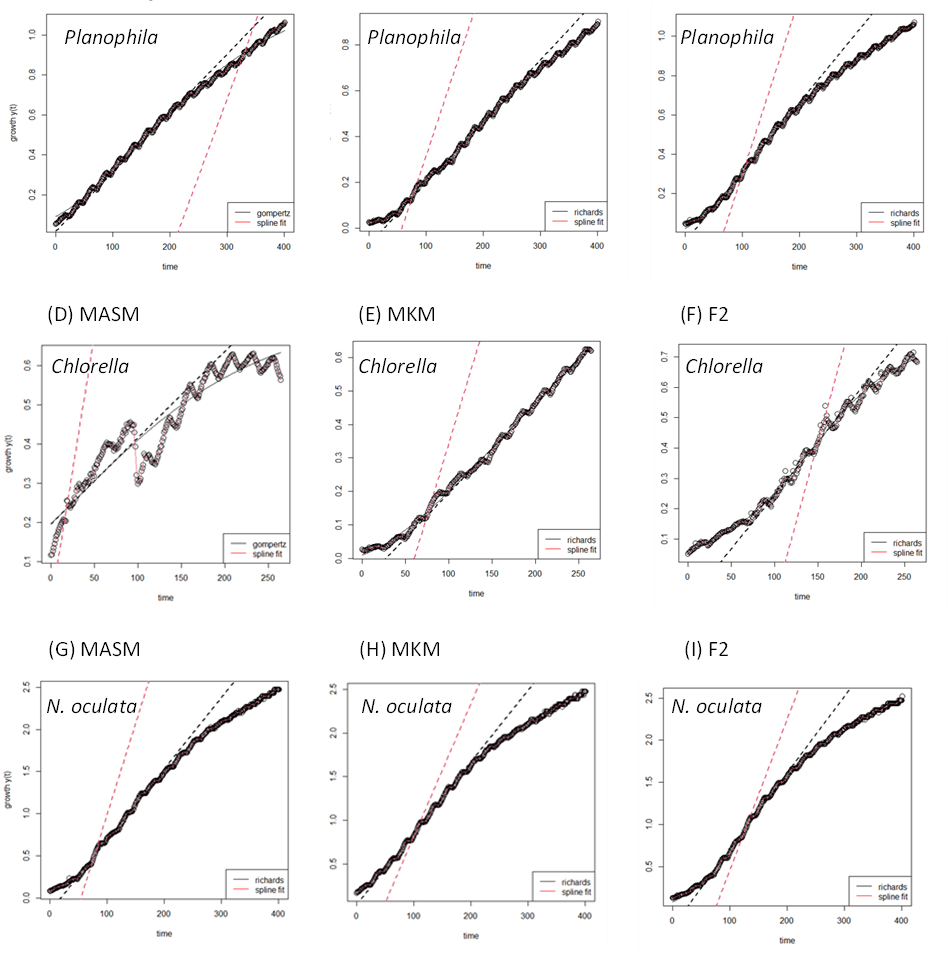


**Figure S2.** The growth (optical density along y -axis) over time (hour along y-axis) of *Planophila* sp. (A, B, C), *Chlorella* sp. (D, E, F) and *N. oculate* (G, H, I) in different media (MASM, MKM, F2) are presented with best fitting parametric model (black colour). Red line indicates spline fit.

**Table S1**. Tukey’s posthoc test to compare the strain for PUFA accumulation

| Strain comparison | Estimate | t value | Probability (>\|t\|) |
| --- | --- | --- | --- |
|  |  |  |  |
| *N. oculata- Chlorella sp.* | 26.282 | 8.488 | < 0.0001 |
| *Planophila sp.- Chlorella sp.* | -19.804 | -6.395 | < 0.0001 |
| *Planophila sp.- Chlorella sp.* | -46.086 | -14.883 | < 0.0001 |

**Table S2**. Tukey’s posthoc test to compare the media for PUFA accumulation

| Media comparison | Estimate | t value | Probability (>\|t\|) |
| --- | --- | --- | --- |
|  |  |  |  |
| MASM-F/2 | -8.115 | -2.621 | 0.0401 |
| MKM-F/2 | 4.840 | 1.563 | 0.2822 |
| MKM-MASM | 12.955 | 4.184 | 0.0011 |
